# Cholera at Buffalo and the West

**Published:** 1849-08

**Authors:** 


*Cholera at Buffalo, and the West.*—The daily reports of the Board of Health in this city, for the past week, show a constant decline in the epidemic. The number of cases reported to the board for the 5th (Sunday,) and 6th insts., was sixty-five. The number of death was twenty-four For the previous two days the number of cases reported was one hundred and ten; of fatal cases, thirty-four. We have reason to hope that the disease has reached its culminating point, and will, ere long, cease its ravages. The whole number of cases reported to the Board up to the 4th instant, inclusive, amounts to 1600; number of deaths, for the same period, 492. The disease, for the most part, has been confined to those who, from insalubrity of situation, bad habits, and social position, are particularly exposed. Some of a different class, however, have fallen victims. The history of the epidemic visitation in this city we shall hope to give, with more detail, at some future time. The disease has prevailed, and still prevails, to some extent, at the village of Black Rock, in the immediate vicinity of Buffalo, and at the County Poor House, situated in Black Rock.

At Cincinati, and St. Louis, the epidemic is nearly extinct.

Sandusky, Ohio, has experienced more suffering from the disease than any other place in the country; indeed, it may be doubted if so great prevalence and mortality have occurred, at any other point, during its march over the world. Among a population of from three to four thousand, reduced by death and flight to not much over one thousand inhabitants, the fatal cases for several days numbered from thirty to forty per diem. Much suffering was experienced at this place from the want of medical attendance. Some of the Physicians of the place died with the disease, and others, we are sorry to say, consulted their own safety, rather than duty, by fleeing with the majority of the population. So soon, however, as the want of medical practitioners was known, Physicians from Cleveland, Cincinati, and other places, were on the ground, to meet the emergency. Among the *corps* from Cleveland was Prof. Ackley, of the Cleveland Medical College. By the latest accounts we learn that the disease had greatly subsided, and, apparently, was about to take its departure.

